# Can a Y Chromosome Degenerate in an Evolutionary Instant? A Commentary on Fong et al. 2023

**DOI:** 10.1093/gbe/evad105

**Published:** 2023-06-08

**Authors:** Deborah Charlesworth, Abigail Hastings, Chay Graham

**Affiliations:** Institute of Ecology and Evolution, School of Biological Sciences, University of Edinburgh, Edinburgh, United Kingdom; Institute of Ecology and Evolution, School of Biological Sciences, University of Edinburgh, Edinburgh, United Kingdom; Institute of Ecology and Evolution, School of Biological Sciences, University of Edinburgh, Edinburgh, United Kingdom

**Keywords:** complete sex linkage, evolutionary strata, dosage compensation, transposable element accumulation

## Abstract

It is well known that the Y chromosomes of Drosophila and mammals and the W chromosomes of birds carry only small fractions of the genes carried by the homologous X or Z chromosomes, and this “genetic degeneration” is associated with loss of recombination between the sex chromosome pair. However, it is still not known how much evolutionary time is needed to reach such nearly complete degeneration. The XY pair of species in a group of closely related poecilid fish is homologous but has been found to have either nondegenerated or completely degenerated Y chromosomes. We evaluate evidence described in a recent paper and show that the available data cast doubt on the view that degeneration has been extraordinarily rapid in the latter (*Micropoecilia* species).

**Significance**

The Y chromosomes of Drosophila and mammals and the W chromosomes of birds are largely nonrecombining and extremely degenerated. Their degeneration can be explained by processes that act following loss of recombination, including Muller's ratchet and interference between selection at different mutant sites, that allow deleterious mutations to accumulate in extensive nonrecombining genome regions carrying many ancestral genes. Evolution of a genetically inert Y chromosome may not only, however, reflect just accumulation of nonfunctional genes but may also involve active reduction of expression of Y-linked genes. Rapid degeneration could then potentially occur. Systems in which the Ys of related species show extreme differences in degeneration are interesting for studies that could support the inference that degeneration has indeed been rapid.

A recent paper suggests that the highly degenerated Y chromosome of a fish might be a case of fast genetic degeneration (Fong et al. 2023). Of the species grouped into the genus *Micropoecilia*, all three species that have been studied (*Micropoecilia picta*, *M. parae*, and *M. bifurca*) have highly degenerated Ys, and as reviewed in Fong et al. (2023), who use the genus name *Poecilia*, *M. picta* and *M. parae* show similar expression of sex-linked genes in both sexes, suggesting that dosage compensation has evolved. In contrast, although the Y chromosome of their close relative, the guppy (*Poecilia reticulata*), is homologous to the *Micropoecilia* species' X, it has not degenerated but carries the same set of genes as the X chromosomes of all these species (fig. 1). These findings suggest that Y-X recombination probably became suppressed in a common ancestor of *Micropoecilia*, but in the guppy the Y occasionally recombines with the X (Winge 1923), preventing degeneration. The new study tested whether the *M. picta* Y stopped recombining since the split from the guppy lineage, and subsequently degenerated, so that less than 10% of the roughly 900 X-linked genes still have Y copies (Charlesworth et al. 2021). Based on small sequence divergence between the Y- and X-linked sequences of such genes, they conclude that loss of recombination and genetic degeneration probably occurred more recently than the split from the guppy lineage and that the alternative suggested by Charlesworth et al. (2021), that there has been a recent turnover even in the guppy lineage, is less likely.

**Fig. 1:**
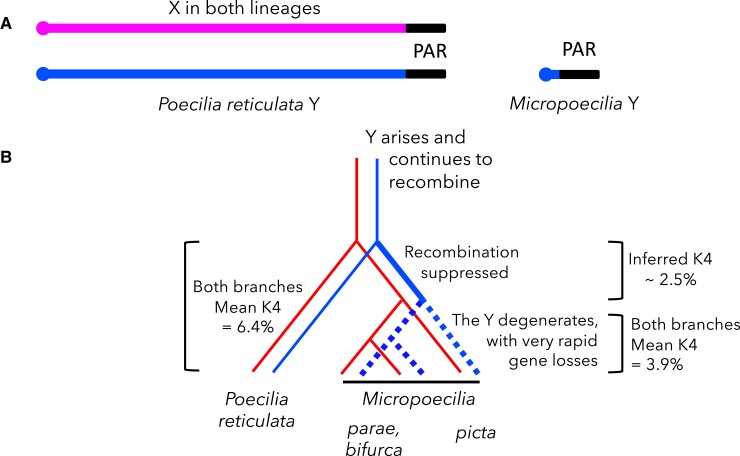
(*A*) Diagram of the *P. reticulata* and *M. picta* XY pairs, showing the small completely Y-linked region in male meiosis in the latter, versus the situation in *P. reticulata*, where the Y is nondegenerated and carries the same genes as the X of both lineages, although it recombines rarely with the X in male meiosis. The diagram shows, roughly to scale, the relative estimated total sizes of the chromosomes. The *M. picta*, only the small Y is illustrated; the X is a similar size to the guppy X, and is about 10 times that of the Y total size (see main text). The PAR is shown in black and is shown as the same sizes on both the Y and X chromosomes. About 10% of the assembly appears to be pseudo-autosomal in the guppy and is similar in both lineages. The size of the completely Y-linked region in *M. picta* is not definitively known. Knowing the relative sizes of the Y-linked region and the PAR, one can predict the values of the TE density within the fully Y-linked region, as explained in the main text. (*B*) Phylogeny of the species with different degrees of genetic degeneration. The thick solid line labelled "recombination suppressed" indicates the lineage during which recombination cessation, genetic degeneration and dosage compensation evolved in the Micropoecilia species ancestor. Neutral sequence divergence is estimated as the nucleotide divergence per 4-fold degenerate site (denoted by K4).

Such rapid and complete degeneration in the *Micropoecilia* lineage seems surprising, but it is important to test whether the evidence supports it, as it is not yet clear how long it might take to evolve complete degeneration and dosage compensation in a system without these properties (such as a system similar to that of the guppy XY pair, which Fong et al. (2023) suggest was the recent ancestral state for both lineages). Theoretical modeling of deleterious mutations following loss of recombination shows that complete linkage hinders the action of natural selection to eliminate deleterious mutations. Modelling of two processes thought to be important, Muller’s ratchet and interference between selection at different mutant sites, can help to understand the likely time course of degeneration (reviewed by Bachtrog 2008). Under biologically plausible mutation rates, a Y-linked region can become highly degenerated in several million generations in a population with an effective population size of 10,000, depending on the number of sites in the nonrecombining genome region at which deleterious mutations can occur; with 2,000 genes, if a single slightly deleterious mutation renders a gene nonfunctional, it takes ∼ 30 million generations for >90% of genes to be lost (Bachtrog 2008). Degeneration is slower with fewer selected sites, and further gene loss after 10% of the ancestral genes have degenerated depends on spread of advantageous mutations and is extremely slow unless mutations are often advantageous. In larger populations, the processes involved earlier in degeneration, Muller's ratchet, and a reduced effective population size due to selection eliminating deleterious mutations (see Charlesworth 1996) are slower, but the final stages are faster. Moreover, the evolution of a genetically inert Y chromosome might involve not just loss of gene functions and of Y-linked genes but also active silencing of Y-linked genes during the evolution of dosage compensation (Charlesworth 1978). Recent modeling suggests a version of such a degeneration process that might potentially occur faster than under mutation accumulation alone if inversions successively suppress recombination in genome regions closely linked to a sex-determining locus; biologically plausible parameters have not, however, yet been modeled (Lenormand and Roze 2022).

Empirical studies of neo-sex chromosome systems in *Drosophila miranda* (reviewed in (Bachtrog 2008; Nozawa et al. 2021) and Lepidoptera (Gu et al. 2017; Gu et al. 2019) have shown that degenerative changes can evolve rapidly after a chromosome carrying many genes instantaneously becomes completely sex-linked, due to fusion with the Y or W chromosome in species with no crossing over in the heterogametic sex. Degeneration will then be as fast as is possible, but it is still incomplete in *D. miranda* (Bachtrog 2006; Nozawa et al. 2021), whose XY synonymous site divergence is about 3% (Bartolomé and Charlesworth 2006). The ancestor of the *M. picta* XY pair (chromosome 12) probably had about 900 genes, like the homologous guppy X chromosome (Künstner et al. 2017), fewer than the *D. miranda* neo-X chromosome, which carries at least functionally distinct 1,800 genes, and as many as 2,700 genes including possible paralogs (Nozawa et al. 2021). Given its smaller number of genes, models of the processes involved in degeneration (Bachtrog 2008) predict that *Micropoecilia* Y should degenerate more slowly than the *D. miranda* neo-Y.

In this new study of *M. picta*, Fong et al. (2023) aimed to test between the two possibilities suggested, that the degenerated Y chromosome is old-established, versus that it evolved rapidly since a recent recombination cessation event is not, however, certain that a recombination suppression event occurred within the guppy lineage. The PAR boundary may alternatively reflect an ancestral Poecilid system that ensures crossover localisation to the terminal regions of the chromosomes (including all Poecilia reticulata autosomes and those of the outgroup species X. maculatus Charlesworth, et al. 2020), so that the boundaries in different species are in similar locations, though perhaps not identical ones. Most *M. picta* sex-linked genes are hemizygous in males, but terminal region genes are found as X–Y gene pairs (Charlesworth et al. 2021). The authors ascertained eight Y–X gene pairs, using SEX-DETector software (Muyle et al. 2016) to classify variants in transcript sequences as fully or partially sex-linked, after genotyping five progeny of each sex from each of four families (the minimum number per family required to reliably identify sex-linked genes). Synonymous site divergence estimates between putative Y and X alleles of these genes were all very low (averaging below 1%). This implies that these genes did not stop recombining before the split from the guppy lineage, since divergence between the species (fig. 1*B*, based on Charlesworth et al. 2021) exceeds 4% for 4-fold degenerate or synonymous sites in seven genes sequenced by Pollux et al. (2014). The authors argue that this suggests differences in the rate of Y chromosome degeneration, slow in *P. reticulata* and *P. wingei*, and rapid evolution of extremely degenerated Y chromosomes and dosage compensation in *M. picta* and *M. parae* following cessation of recombination since the split from the *Poecilia* lineage.

However, guppy homologs of 7 of the 8 *M. picta* genes with Y-linked copies are distal to 25 Mb in the *M. picta* sex chromosome assembly, which is where the pseudo-autosomal region, or PAR, is found in both M. picta and the guppy (figs. 2 of Fong et al. 2023 and Charlesworth et al. 2021, which also shows some genes near 10 Mb reflecting misassembly of some autosomal genes, which Fong et al. correctly classified as non-sex-linked). Those genes analyzed by Fong et al. could therefore be within a physically small terminal PAR, which still recombines and has not degenerated (but not classified by Fong et al. as PAR genes because the small families analyzed included no recombinants). Alternatively, they could be within a recently formed completely sex-linked “evolutionary stratum”; two genes that are pseudo-autosomal in the guppy and *M. parae* indeed appear to be fully sex-linked in *M. picta* (Charlesworth et al. 2021). This resembles recent changes in the PAR boundary detected in humans, long after most of the Y became nonrecombining (Van Laere et al. 2008), and in the plant genus *Silene*, where one region recently became fully Y-linked in *Silene latifolia* but not in its close relative *S. dioica* (Campos et al. 2017; Filatov 2022). Consistent with the possibility that these genes became Y-linked recently, Fong et al.'s phylogenetic analysis groups *Micropoecilia* XY sequence pairs together, separate from their guppy orthologs. A location in a new evolutionary stratum near the PAR can reconcile the low Y–X divergence of these *M. picta* genes with the much higher divergence value between *M. picta* and other *Micropoecilia* species (fig. 1*B*). If Y-linkage evolved in a *Micropoecilia* ancestor of *M. picta* and *M. parae* (as Fong et al.'s finding of many shared Y-specific sequences supports), Y–X divergence within both species should be at least as high as between-species divergence, which is not the case (fig. 1*B*). The divergence value of less than 1% estimated by Fong et al. (2023) for their set of *M. picta* genes does support their conclusion that recombination for these Y-linked genes was halted recently, but this cannot be extended to infer “similar timing of recombination loss between the X and Y chromosomes in these two species” for the rest of the *M. picta* Y. Indeed Fong et al. explicitly note that, consistent with (Charlesworth et al. 2021), informative sequences are lacking across most of the *M. picta* Y and that much of the Y is probably deleted, explaining the small cytological size of the *M. picta* Y (Nanda et al. 2022).

The authors also used the abundance of k-mers identified by a now-standard approach proposed by Carvalho and Clark (2013) for discovering male-specific (and thus Y-linked) sequences, to evaluate the time since the *M. picta* Y stopped recombining with the X. They detected only a modest amount of such k-mers and suggest that this indicates only a short time since repetitive sequences started to accumulate. However, a small representation of male-specific sequences could reflect either a small enrichment on the Y chromosome that stopped recombining recently or a great enrichment in physically small region. Fong et al. (2023) estimated the size of the entire Y to be about 2.6 Mb, roughly one tenth of the size of the X, consistent with the small cytological size of the *M. picta* Y (Nanda et al. 2022). The portion that represents the PAR, versus completely Y-linked regions, was estimated from the number of genes with diploid coverage in *M. picta* males to be less than about 10% of all chromosome 12 genes (Charlesworth et al. 2021), which is consistent with a small paired region in male meiosis (fig. 3B in Nanda et al. 2022). In the guppy, the PAR occupies around 10% of the roughly 26.7 Mb chromosome 12 (Bergero et al. 2019; Charlesworth et al. 2020). This is similar to the size estimated for the entire *M. picta* Y, which may thus consist almost completely of PAR sequences, plus a physically small and extremely degenerated fully Y-linked region that is mainly repetitive.

We analyzed repetitive sequence density in *M. picta*, as this can be informative about the Y-linked region. We detected a consistently higher density of most types of transposable elements on the sex chromosomes, compared with the genomic average, though the overall difference is small (about 30.8% vs. 29.2%, respectively, see details in fig. 2). This value for the entire XY pair will largely reflect the density on the much larger X chromosome (including the PAR); thus, even the small difference observed suggests a considerably higher density for the small fully Y-linked region.

**Fig. 2:**
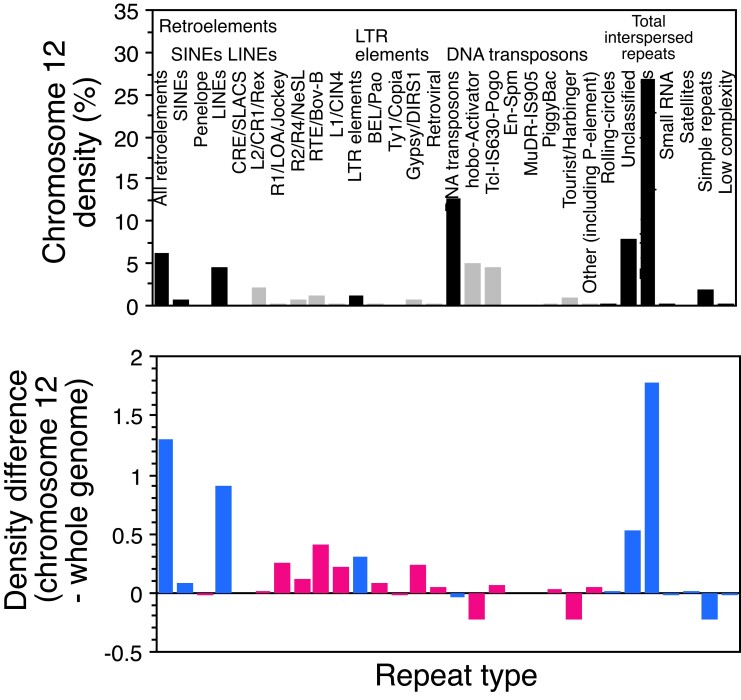
Estimated transposable element and other repeat densities in the assembly of a male *M. picta* individual. The top part shows the genome-wide density estimates for different types of repeats, and the bottom part shows the estimated differences between chromosome 12 and the genomic means; both parts show differences for broad categories of repeats (indicated in the upper part), and for more specific types, which are labeled in the upper part (grey columns in that part). The results were obtained by RepeatMasker and RepeatModeler analyses (Flynn et al. 2020) of the *M. picta* male genome assembly (Charlesworth et al. 2021).

Using the estimated *M. picta* genome-wide density (denoted by *r0*) and assuming a similar density for the X and the PAR, one can roughly estimate the proportion of the Y that is completely Y-linked (denoted here by *d*), as follows. Given that the entire Y is about 10% of the X size, the expected density for the sex chromosome pair, *rXY*, is *r0* + 0.1 × [(1−*d*) × *r0* + *d* × *r*1]/1.1. Assuming an overall mean *r0* estimate and an observed *Rxy* value, this formula can then be applied to predict *r*1 for any value of *d*; if *d* = 0.25 (as in fig. 1*A*), this predicts an *r*1 value of 98%, in other words a completely degenerated region with few sequences other than repeats. It remains to be determined whether 0.25 is a plausible value for *d*, but higher values would still correspond to considerable enrichment of repeats in the completely Y-linked region, readily accounting for a small number of male-specific k-mers.

In addition, the small cytological size of the *M. picta* Y probably reflects deletion(s) of large parts of the chromosome (as Fong et al. note). This could occur after genes had lost functions, which would also be likely to delete repetitive sequences, reducing number of male-specific k-mers. If large amounts of repetitive sequences have been deleted, the density in the present fully Y-linked region would underestimate the true accumulation of such sequences, and the time since recombination stopped, to an unknown extent. It therefore seems premature to conclude that the *M. picta* Y stopped recombining, and started to degenerate, very recently.

These species may nevertheless help to understand the time-course of recombination loss and genetic degeneration. The hypothesis that the Y stopped recombining in a common *Micropoecilia* ancestor, before the split of the extant species (fig. 1*B*), suggests that this time interval was sufficient for recombination to stop across almost the entire chromosome 12 (except for the PAR and small set of genes in the *M. picta*-specific stratum noted above).

Even under the recently proposed models for recombination suppression (Jeffries et al. 2021; Jay et al. 2022; Lenormand and Roze 2022) that the authors suggest might have been involved in these fish, it is not yet clear that this time would be long enough for most of the Y to become nonrecombining, as multiple events, each suppressing recombination across a part of the region, are involved. Overall, the possibility that the Y chromosome's lack of crossing-over in *Micropoecilia* species dates from before the split from the *Poecilia* lineage (Charlesworth et al. 2021) cannot yet be ruled out.

## Data Availability

The RepeatModeler output file will be deposited in Dryad.
